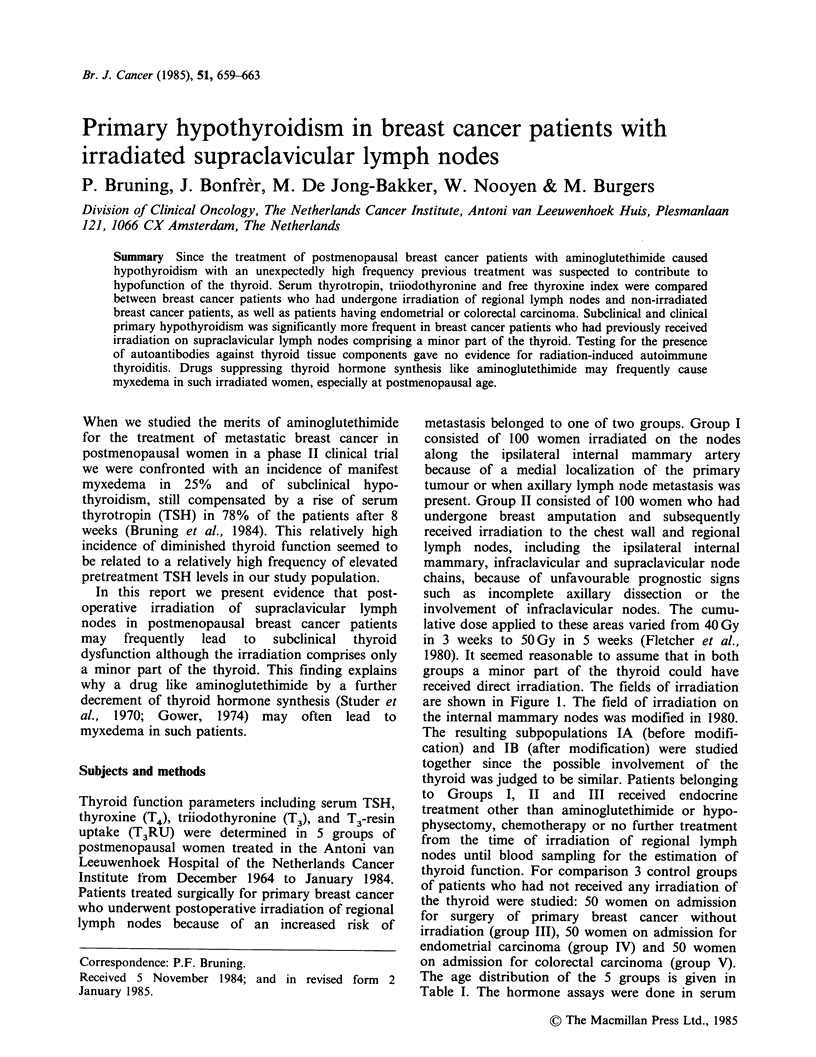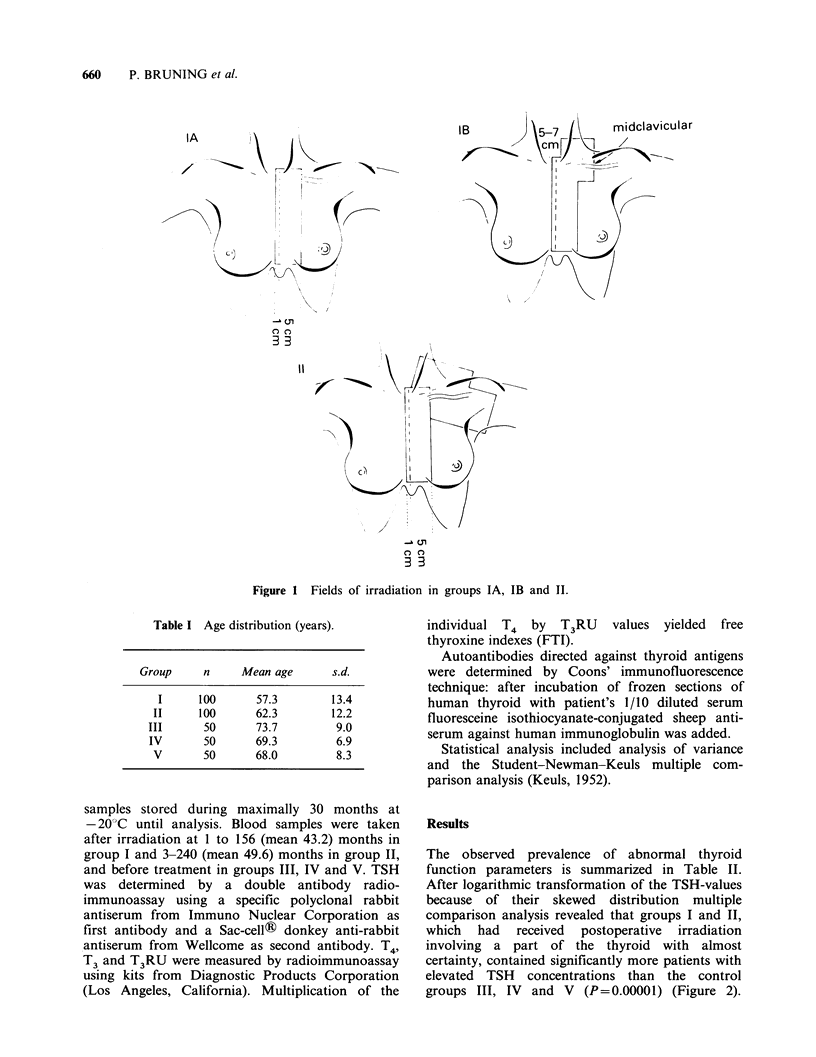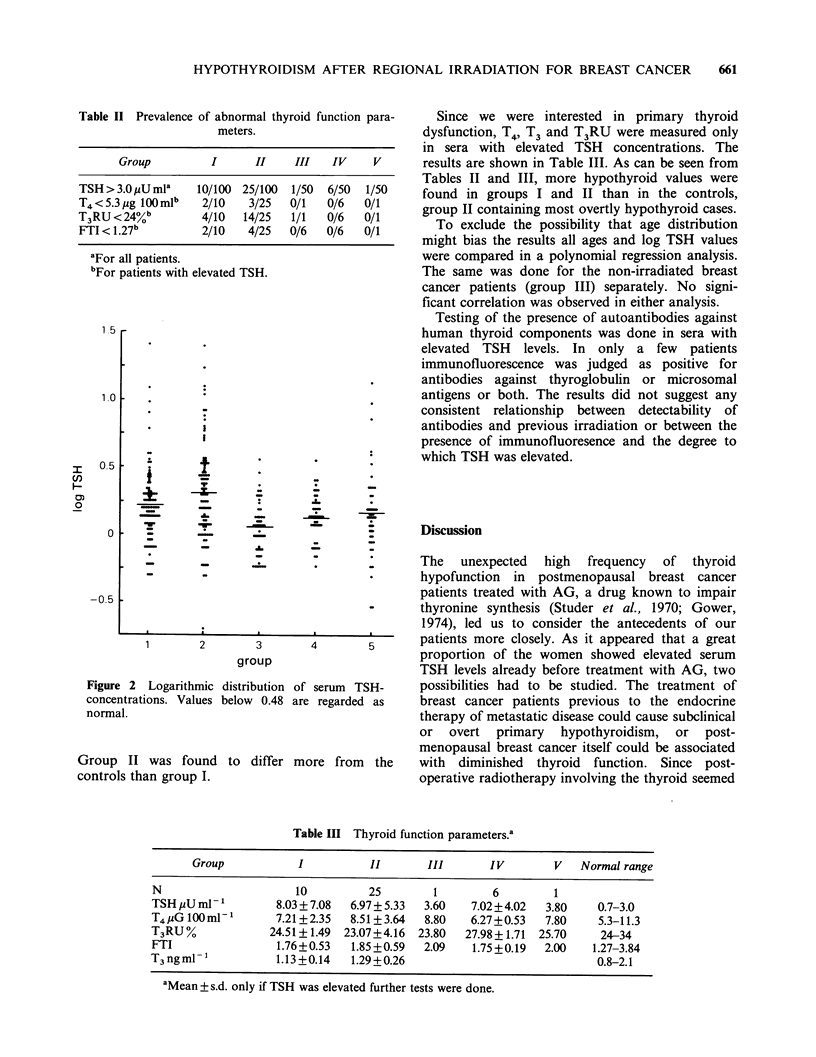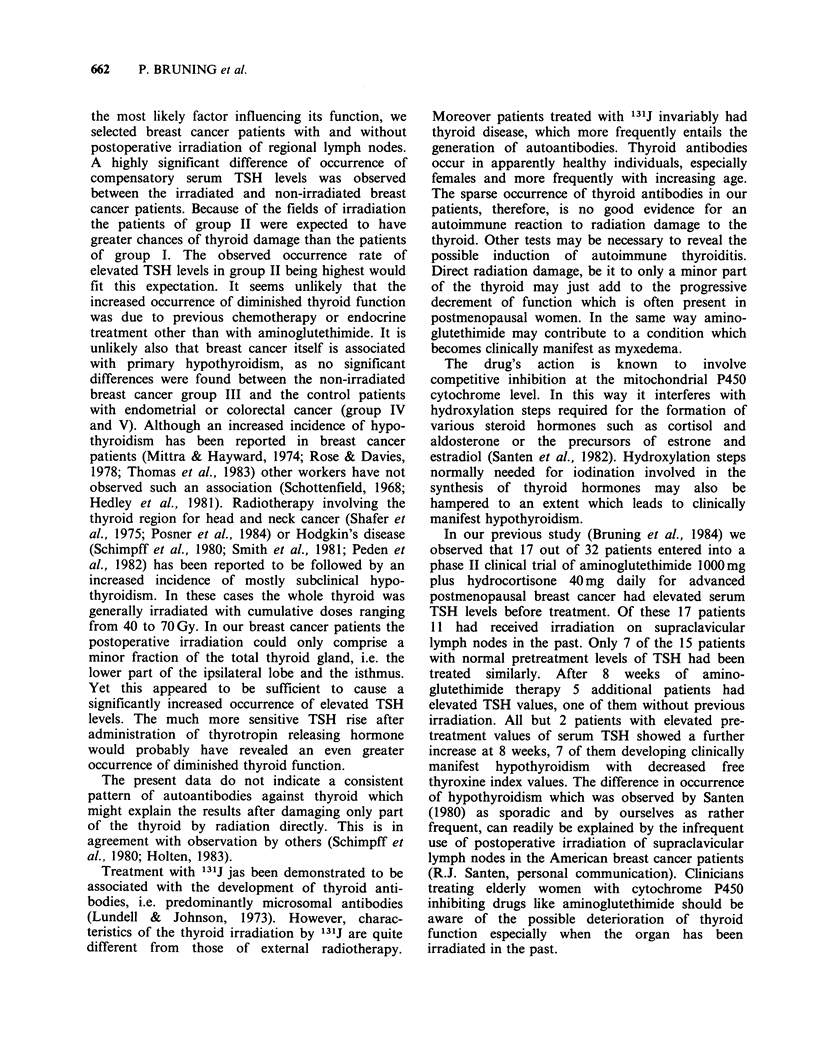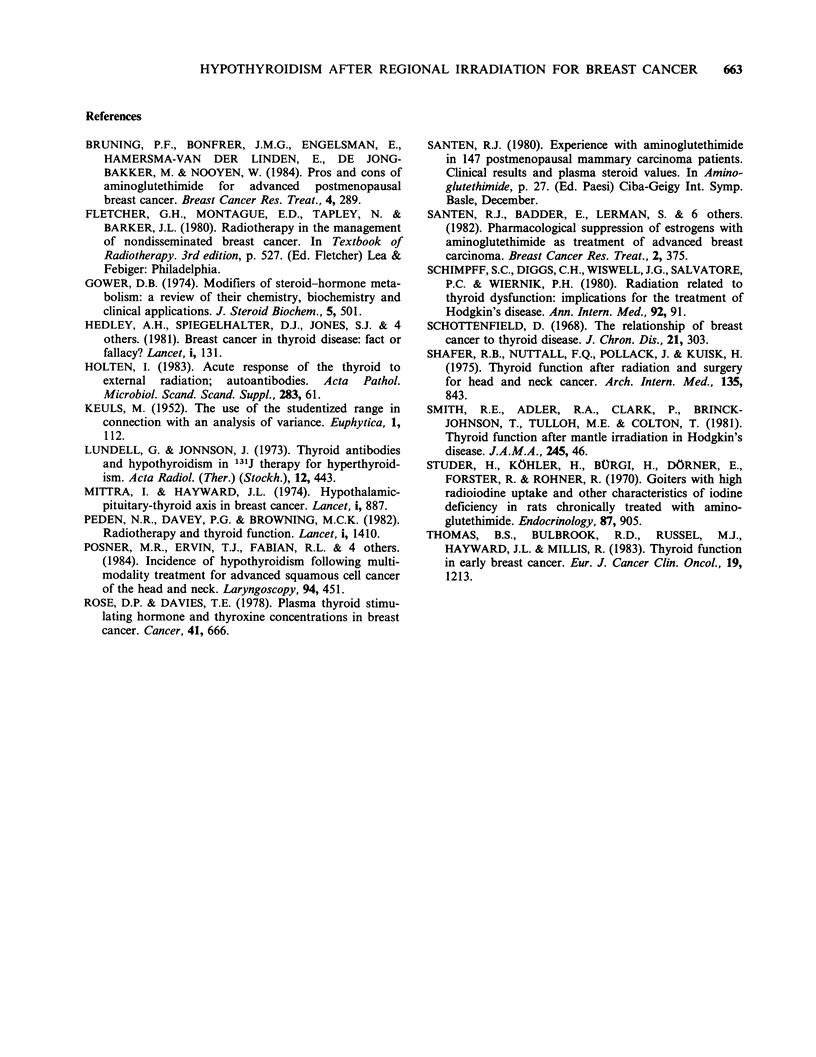# Primary hypothyroidism in breast cancer patients with irradiated supraclavicular lymph nodes.

**DOI:** 10.1038/bjc.1985.99

**Published:** 1985-05

**Authors:** P. Bruning, J. Bonfrèr, M. De Jong-Bakker, W. Nooyen, M. Burgers

## Abstract

Since the treatment of postmenopausal breast cancer patients with aminoglutethimide caused hypothyroidism with an unexpectedly high frequency previous treatment was suspected to contribute to hypofunction of the thyroid. Serum thyrotropin, triiodothyronine and free thyroxine index were compared between breast cancer patients who had undergone irradiation of regional lymph nodes and non-irradiated breast cancer patients, as well as patients having endometrial or colorectal carcinoma. Subclinical and clinical primary hypothyroidism was significantly more frequent in breast cancer patients who had previously received irradiation on supraclavicular lymph nodes comprising a minor part of the thyroid. Testing for the presence of autoantibodies against thyroid tissue components gave no evidence for radiation-induced autoimmune thyroiditis. Drugs suppressing thyroid hormone synthesis like aminoglutethimide may frequently cause myxedema in such irradiated women, especially at postmenopausal age.


*Br. J. Cancer* (1985), **51**, 659–663

### Primary hypothyroidism in breast cancer patients with irradiated supraclavicular lymph nodes

P. Bruning, J. Bonfrèr, M. De Jong-Bakker, W. Nooyen & M. Burgers

*Division of Clinical Oncology, The Netherlands Cancer Institute, Antoni van Leeuwenhoek Huis, Plesmanlaan 121, 1066 CX Amsterdam, The Netherlands*

**Summary** Since the treatment of postmenopausal breast cancer patients with aminoglutethimide caused hypothyroidism with an unexpectedly high frequency previous treatment was suspected to contribute to hypofunction of the thyroid. Serum thyrotropin, triiodothyronine and free thyroxine index were compared between breast cancer patients who had undergone irradiation of regional lymph nodes and non-irradiated breast cancer patients, as well as patients having endometrial or colorectal carcinoma. Subclinical and clinical primary hypothyroidism was significantly more frequent in breast cancer patients who had previously received irradiation on supraclavicular lymph nodes comprising a minor part of the thyroid. Testing for the presence of autoantibodies against thyroid tissue components gave no evidence for radiation-induced autoimmune thyroiditis. Drugs suppressing thyroid hormone synthesis like aminoglutethimide may frequently cause myxedema in such irradiated women, especially at postmenopausal age.

When we studied the merits of aminoglutethimide for the treatment of metastatic breast cancer in postmenopausal women in a phase II clinical trial we were confronted with an incidence of manifest myxedema in 25% and of subclinical hypothyroidism, still compensated by a rise of serum thyrotropin (TSH) in 78% of the patients after 8 weeks (Bruning *et al.*, 1984). This relatively high incidence of diminished thyroid function seemed to be related to a relatively high frequency of elevated pretreatment TSH levels in our study population.

In this report we present evidence that postoperative irradiation of supraclavicular lymph nodes in postmenopausal breast cancer patients may frequently lead to subclinical thyroid dysfunction although the irradiation comprises only a minor part of the thyroid. This finding explains why a drug like aminoglutethimide by a further decrement of thyroid hormone synthesis (Studer *et al.*, 1970; Gower, 1974) may often lead to myxedema in such patients.

### Subjects and methods

Thyroid function parameters including serum TSH, thyroxine ($T_4$), triiodothyronine ($T_3$), and $T_3$-resin uptake ($T_3RU$) were determined in 5 groups of postmenopausal women treated in the Antoni van Leeuwenhoek Hospital of the Netherlands Cancer Institute from December 1964 to January 1984. Patients treated surgically for primary breast cancer who underwent postoperative irradiation of regional lymph nodes because of an increased risk of metastasis belonged to one of two groups. Group I consisted of 100 women irradiated on the nodes along the ipsilateral internal mammary artery because of a medial localization of the primary tumour or when axillary lymph node metastasis was present. Group II consisted of 100 women who had undergone breast amputation and subsequently received irradiation to the chest wall and regional lymph nodes, including the ipsilateral internal mammary, infraclavicular and supraclavicular node chains, because of unfavourable prognostic signs such as incomplete axillary dissection or the involvement of infraclavicular nodes. The cumulative dose applied to these areas varied from 40 Gy in 3 weeks to 50 Gy in 5 weeks (Fletcher *et al.*, 1980). It seemed reasonable to assume that in both groups a minor part of the thyroid could have received direct irradiation. The fields of irradiation are shown in Figure 1. The field of irradiation on the internal mammary nodes was modified in 1980. The resulting subpopulations IA (before modification) and IB (after modification) were studied together since the possible involvement of the thyroid was judged to be similar. Patients belonging to Groups I, II and III received endocrine treatment other than aminoglutethimide or hypophysectomy, chemotherapy or no further treatment from the time of irradiation of regional lymph nodes until blood sampling for the estimation of thyroid function. For comparison 3 control groups of patients who had not received any irradiation of the thyroid were studied: 50 women on admission for surgery of primary breast cancer without irradiation (group III), 50 women on admission for endometrial carcinoma (group IV) and 50 women on admission for colorectal carcinoma (group V). The age distribution of the 5 groups is given in Table I. The hormone assays were done in serum

Correspondence: P.F. Bruning.

Received 5 November 1984; and in revised form 2 January 1985.

© The Macmillan Press Ltd., 1985

**Figure 1** Fields of irradiation in groups IA, IB and II.

**Table I** Age distribution (years).

| *Group* | *n* | *Mean age* | *s.d.* |
|---|---|---|---|
| I | 100 | 57.3 | 13.4 |
| II | 100 | 62.3 | 12.2 |
| III | 50 | 73.7 | 9.0 |
| IV | 50 | 69.3 | 6.9 |
| V | 50 | 68.0 | 8.3 |

samples stored during maximally 30 months at $-20^\circ$C until analysis. Blood samples were taken after irradiation at 1 to 156 (mean 43.2) months in group I and 3–240 (mean 49.6) months in group II, and before treatment in groups III, IV and V. TSH was determined by a double antibody radioimmunoassay using a specific polyclonal rabbit antiserum from Immuno Nuclear Corporation as first antibody and a Sac-cell® donkey anti-rabbit antiserum from Wellcome as second antibody. $T_4$, $T_3$ and $T_3$RU were measured by radioimmunoassay using kits from Diagnostic Products Corporation (Los Angeles, California). Multiplication of the individual $T_4$ by $T_3$RU values yielded free thyroxine indexes (FTI).

Autoantibodies directed against thyroid antigens were determined by Coons' immunofluorescence technique: after incubation of frozen sections of human thyroid with patient's 1/10 diluted serum fluoresceine isothiocyanate-conjugated sheep antiserum against human immunoglobulin was added.

Statistical analysis included analysis of variance and the Student–Newman–Keuls multiple comparison analysis (Keuls, 1952).

### Results

The observed prevalence of abnormal thyroid function parameters is summarized in Table II. After logarithmic transformation of the TSH-values because of their skewed distribution multiple comparison analysis revealed that groups I and II, which had received postoperative irradiation involving a part of the thyroid with almost certainty, contained significantly more patients with elevated TSH concentrations than the control groups III, IV and V ($P$=0.00001) (Figure 2).

**Table II** Prevalence of abnormal thyroid function parameters.

| *Group* | *I* | *II* | *III* | *IV* | *V* |
|---|---|---|---|---|---|
| TSH > 3.0 $\mu$U ml[a] | 10/100 | 25/100 | 1/50 | 6/50 | 1/50 |
| $T_4$ < 5.3 $\mu$g 100 ml[b] | 2/10 | 3/25 | 0/1 | 0/6 | 0/1 |
| $T_3$RU < 24%[b] | 4/10 | 14/25 | 1/1 | 0/6 | 0/1 |
| FTI < 1.27[b] | 2/10 | 4/25 | 0/6 | 0/6 | 0/1 |

[a]For all patients.
[b]For patients with elevated TSH.

**Figure 2** Logarithmic distribution of serum TSH-concentrations. Values below 0.48 are regarded as normal.

Group II was found to differ more from the controls than group I.

Since we were interested in primary thyroid dysfunction, $T_4$, $T_3$ and $T_3$RU were measured only in sera with elevated TSH concentrations. The results are shown in Table III. As can be seen from Tables II and III, more hypothyroid values were found in groups I and II than in the controls, group II containing most overtly hypothyroid cases.

To exclude the possibility that age distribution might bias the results all ages and log TSH values were compared in a polynomial regression analysis. The same was done for the non-irradiated breast cancer patients (group III) separately. No significant correlation was observed in either analysis.

Testing of the presence of autoantibodies against human thyroid components was done in sera with elevated TSH levels. In only a few patients immunofluorescence was judged as positive for antibodies against thyroglobulin or microsomal antigens or both. The results did not suggest any consistent relationship between detectability of antibodies and previous irradiation or between the presence of immunofluoresence and the degree to which TSH was elevated.

### Discussion

The unexpected high frequency of thyroid hypofunction in postmenopausal breast cancer patients treated with AG, a drug known to impair thyronine synthesis (Studer *et al.*, 1970; Gower, 1974), led us to consider the antecedents of our patients more closely. As it appeared that a great proportion of the women showed elevated serum TSH levels already before treatment with AG, two possibilities had to be studied. The treatment of breast cancer patients previous to the endocrine therapy of metastatic disease could cause subclinical or overt primary hypothyroidism, or postmenopausal breast cancer itself could be associated with diminished thyroid function. Since postoperative radiotherapy involving the thyroid seemed

**Table III** Thyroid function parameters.[a]

| *Group* | *I* | *II* | *III* | *IV* | *V* | *Normal range* |
|---|---|---|---|---|---|---|
| N | 10 | 25 | 1 | 6 | 1 | |
| TSH $\mu$U ml$^{-1}$ | 8.03 ± 7.08 | 6.97 ± 5.33 | 3.60 | 7.02 ± 4.02 | 3.80 | 0.7–3.0 |
| $T_4$ $\mu$G 100 ml$^{-1}$ | 7.21 ± 2.35 | 8.51 ± 3.64 | 8.80 | 6.27 ± 0.53 | 7.80 | 5.3–11.3 |
| $T_3$RU % | 24.51 ± 1.49 | 23.07 ± 4.16 | 23.80 | 27.98 ± 1.71 | 25.70 | 24–34 |
| FTI | 1.76 ± 0.53 | 1.85 ± 0.59 | 2.09 | 1.75 ± 0.19 | 2.00 | 1.27–3.84 |
| $T_3$ ng ml$^{-1}$ | 1.13 ± 0.14 | 1.29 ± 0.26 | | | | 0.8–2.1 |

[a]Mean ± s.d. only if TSH was elevated further tests were done.

the most likely factor influencing its function, we selected breast cancer patients with and without postoperative irradiation of regional lymph nodes. A highly significant difference of occurrence of compensatory serum TSH levels was observed between the irradiated and non-irradiated breast cancer patients. Because of the fields of irradiation the patients of group II were expected to have greater chances of thyroid damage than the patients of group I. The observed occurrence rate of elevated TSH levels in group II being highest would fit this expectation. It seems unlikely that the increased occurrence of diminished thyroid function was due to previous chemotherapy or endocrine treatment other than with aminoglutethimide. It is unlikely also that breast cancer itself is associated with primary hypothyroidism, as no significant differences were found between the non-irradiated breast cancer group III and the control patients with endometrial or colorectal cancer (group IV and V). Although an increased incidence of hypothyroidism has been reported in breast cancer patients (Mittra & Hayward, 1974; Rose & Davies, 1978; Thomas *et al.*, 1983) other workers have not observed such an association (Schottenfield, 1968; Hedley *et al.*, 1981). Radiotherapy involving the thyroid region for head and neck cancer (Shafer *et al.*, 1975; Posner *et al.*, 1984) or Hodgkin's disease (Schimpff *et al.*, 1980; Smith *et al.*, 1981; Peden *et al.*, 1982) has been reported to be followed by an increased incidence of mostly subclinical hypothyroidism. In these cases the whole thyroid was generally irradiated with cumulative doses ranging from 40 to 70 Gy. In our breast cancer patients the postoperative irradiation could only comprise a minor fraction of the total thyroid gland, i.e. the lower part of the ipsilateral lobe and the isthmus. Yet this appeared to be sufficient to cause a significantly increased occurrence of elevated TSH levels. The much more sensitive TSH rise after administration of thyrotropin releasing hormone would probably have revealed an even greater occurrence of diminished thyroid function.

The present data do not indicate a consistent pattern of autoantibodies against thyroid which might explain the results after damaging only part of the thyroid by radiation directly. This is in agreement with observation by others (Schimpff *et al.*, 1980; Holten, 1983).

Treatment with $^{131}J$ jas been demonstrated to be associated with the development of thyroid antibodies, i.e. predominantly microsomal antibodies (Lundell & Johnson, 1973). However, characteristics of the thyroid irradiation by $^{131}J$ are quite different from those of external radiotherapy. Moreover patients treated with $^{131}J$ invariably had thyroid disease, which more frequently entails the generation of autoantibodies. Thyroid antibodies occur in apparently healthy individuals, especially females and more frequently with increasing age. The sparse occurrence of thyroid antibodies in our patients, therefore, is no good evidence for an autoimmune reaction to radiation damage to the thyroid. Other tests may be necessary to reveal the possible induction of autoimmune thyroiditis. Direct radiation damage, be it to only a minor part of the thyroid may just add to the progressive decrement of function which is often present in postmenopausal women. In the same way aminoglutethimide may contribute to a condition which becomes clinically manifest as myxedema.

The drug's action is known to involve competitive inhibition at the mitochondrial P450 cytochrome level. In this way it interferes with hydroxylation steps required for the formation of various steroid hormones such as cortisol and aldosterone or the precursors of estrone and estradiol (Santen *et al.*, 1982). Hydroxylation steps normally needed for iodination involved in the synthesis of thyroid hormones may also be hampered to an extent which leads to clinically manifest hypothyroidism.

In our previous study (Bruning *et al.*, 1984) we observed that 17 out of 32 patients entered into a phase II clinical trial of aminoglutethimide 1000 mg plus hydrocortisone 40 mg daily for advanced postmenopausal breast cancer had elevated serum TSH levels before treatment. Of these 17 patients 11 had received irradiation on supraclavicular lymph nodes in the past. Only 7 of the 15 patients with normal pretreatment levels of TSH had been treated similarly. After 8 weeks of aminoglutethimide therapy 5 additional patients had elevated TSH values, one of them without previous irradiation. All but 2 patients with elevated pretreatment values of serum TSH showed a further increase at 8 weeks, 7 of them developing clinically manifest hypothyroidism with decreased free thyroxine index values. The difference in occurrence of hypothyroidism which was observed by Santen (1980) as sporadic and by ourselves as rather frequent, can readily be explained by the infrequent use of postoperative irradiation of supraclavicular lymph nodes in the American breast cancer patients (R.J. Santen, personal communication). Clinicians treating elderly women with cytochrome P450 inhibiting drugs like aminoglutethimide should be aware of the possible deterioration of thyroid function especially when the organ has been irradiated in the past.

**References**

BRUNING, P.F., BONFRER, J.M.G., ENGELSMAN, E., HAMERSMA-VAN DER LINDEN, E., DE JONG-BAKKER, M. & NOOYEN, W. (1984). Pros and cons of aminoglutethimide for advanced postmenopausal breast cancer. *Breast Cancer Res. Treat.*, **4**, 289.

FLETCHER, G.H., MONTAGUE, E.D., TAPLEY, N. & BARKER, J.L. (1980). Radiotherapy in the management of nondisseminated breast cancer. In *Textbook of Radiotherapy. 3rd edition*, p. 527. (Ed. Fletcher) Lea & Febiger: Philadelphia.

GOWER, D.B. (1974). Modifiers of steroid–hormone metabolism: a review of their chemistry, biochemistry and clinical applications. *J. Steroid Biochem.*, **5**, 501.

HEDLEY, A.H., SPIEGELHALTER, D.J., JONES, S.J. & 4 others. (1981). Breast cancer in thyroid disease: fact or fallacy? *Lancet*, **i**, 131.

HOLTEN, I. (1983). Acute response of the thyroid to external radiation; autoantibodies. *Acta Pathol. Microbiol. Scand. Scand. Suppl.*, **283**, 61.

KEULS, M. (1952). The use of the studentized range in connection with an analysis of variance. *Euphytica*, **1**, 112.

LUNDELL, G. & JONNSON, J. (1973). Thyroid antibodies and hypothyroidism in $^{131}$J therapy for hyperthyroidism. *Acta Radiol.* (*Ther.*) (*Stockh.*), **12**, 443.

MITTRA, I. & HAYWARD, J.L. (1974). Hypothalamic-pituitary-thyroid axis in breast cancer. *Lancet*, **i**, 887.

PEDEN, N.R., DAVEY, P.G. & BROWNING, M.C.K. (1982). Radiotherapy and thyroid function. *Lancet*, **i**, 1410.

POSNER, M.R., ERVIN, T.J., FABIAN, R.L. & 4 others. (1984). Incidence of hypothyroidism following multimodality treatment for advanced squamous cell cancer of the head and neck. *Laryngoscopy*, **94**, 451.

ROSE, D.P. & DAVIES, T.E. (1978). Plasma thyroid stimulating hormone and thyroxine concentrations in breast cancer. *Cancer*, **41**, 666.

SANTEN, R.J. (1980). Experience with aminoglutethimide in 147 postmenopausal mammary carcinoma patients. Clinical results and plasma steroid values. In *Aminoglutethimide*, p. 27. (Ed. Paesi) Ciba-Geigy Int. Symp. Basle, December.

SANTEN, R.J., BADDER, E., LERMAN, S. & 6 others. (1982). Pharmacological suppression of estrogens with aminoglutethimide as treatment of advanced breast carcinoma. *Breast Cancer Res. Treat.*, **2**, 375.

SCHIMPFF, S.C., DIGGS, C.H., WISWELL, J.G., SALVATORE, P.C. & WIERNIK, P.H. (1980). Radiation related to thyroid dysfunction: implications for the treatment of Hodgkin's disease. *Ann. Intern. Med.*, **92**, 91.

SCHOTTENFIELD, D. (1968). The relationship of breast cancer to thyroid disease. *J. Chron. Dis.*, **21**, 303.

SHAFER, R.B., NUTTALL, F.Q., POLLACK, J. & KUISK, H. (1975). Thyroid function after radiation and surgery for head and neck cancer. *Arch. Intern. Med.*, **135**, 843.

SMITH, R.E., ADLER, R.A., CLARK, P., BRINCK-JOHNSON, T., TULLOH, M.E. & COLTON, T. (1981). Thyroid function after mantle irradiation in Hodgkin's disease. *J.A.M.A.*, **245**, 46.

STUDER, H., KÖHLER, H., BÜRGI, H., DÖRNER, E., FORSTER, R. & ROHNER, R. (1970). Goiters with high radioiodine uptake and other characteristics of iodine deficiency in rats chronically treated with aminoglutethimide. *Endocrinology*, **87**, 905.

THOMAS, B.S., BULBROOK, R.D., RUSSEL, M.J., HAYWARD, J.L. & MILLIS, R. (1983). Thyroid function in early breast cancer. *Eur. J. Cancer Clin. Oncol.*, **19**, 1213.